# Modified lung ultrasound score predicts ventilation requirements in neonatal respiratory distress syndrome

**DOI:** 10.1186/s12887-020-02485-z

**Published:** 2021-01-06

**Authors:** Piotr Szymański, Piotr Kruczek, Roman Hożejowski, Piotr Wais

**Affiliations:** 1 Department of Pediatrics, Jagiellonian University Medical College, Cracow, Poland; 2 Present address: Department of Neonatology, Ujastek Medical Center, ul. Ujastek 3, 31-752 Cracow, Poland; 3 Medical Department, Chiesi Poland, Warsaw, Poland; 4 Department of Informatics, Carpathian State University in Krosno, Krosno, Poland

**Keywords:** Lung ultrasound, Respiratory distress syndrome, Neonate, Ventilation

## Abstract

**Background:**

We propose a modified lung ultrasound (LUS) score in neonates with respiratory distress syndrome (RDS), which includes posterior instead of lateral lung fields, and a 5-grade rating scale instead of a 4-grade rating scale. The purpose of this study was to evaluate the reproducibility of the rating scale and its correlation with blood oxygenation and to assess the ability of early post-birth scans to predict the mode of respiratory support on day of life 3 (DOL 3). As a secondary objective, the weight of posterior scans in the overall LUS score was assessed.

**Methods:**

We analyzed 619 serial lung scans performed in 70 preterm infants < 32 weeks gestation and birth weight < 1500 g. Assessments were performed within 24 h of birth (LUS_0_) and on days 2, 3, 5, 7, 10, 14, 21 and 28. LUS scores were correlated with oxygen saturation over fraction of inspired oxygen (S/F) and mode of respiratory support. Interrater agreement was determined with the intraclass correlation coefficient (ICC) and Cronbach’s alpha. Probabilities of the need for various respiratory support modes on DOL 3 were assessed with ordinal logistic regression. Least square (ls) means of the posterior and anterior pulmonary field scores were compared.

**Results:**

The LUS score correlated significantly with S/F (Spearman rho = −0.635; *p* < 0.0001) and had excellent interrater agreement (ICC = 0.94, 95% CI 0.93–0.95; Cronbach’s alpha = 0.99). Significant predictors of ventilation requirements on DOL 3 were LUS_0_ (*p* < 0.016) and birth weight (BW) (*p* < 0.001). In the ROC analysis, LUS_0_ had high reliability in prognosing invasive ventilation on DOL 3 (AUC = 0.845 (95% DeLong CI: 0.738–0.951; *p* < 0.001)). Invasive ventilation was the most likely mode of respiratory support for LUS_0_ scores: ≥7 (in infants with BW 900 g), ≥ 10 (in infants with BW 1050 g) and ≥ 15 (in infants with BW 1280 g). Posterior fields exhibited significantly higher average scores than anterior fields. Respective ls means (confidence levels) were 4.0 (3.8–4.1) vs. 2.2 (2.0–2.4); *p* < 0.001.

**Conclusions:**

Post-birth LUS predicts ventilation requirements on DOL 3. Scores of posterior pulmonary fields have a predominant weight in the overall LUS score.

## Background

An increasing number of reports have recently been published on the usefulness of lung ultrasound (LUS) to assess lung function in premature infants with respiratory distress syndrome (RDS) [1–7]. In the reports concerning LUS score in neonatal RDS, sonograms were limited to the early post-birth period, typically the first 24 h of life [1–6]. These early post-birth LUS scores proved to be well correlated with the gas exchange indices [1, 6]. In some studies, LUS scores also had a significant prognostic value relative to the short-term endpoint, e.g., the need for exogenous surfactant [1, 2, 5, 6].

The scoring scheme as described initially by the group of DeLuca [6] was based on evaluation of anterior and lateral pulmonary fields. Anterior fields were additionally divided into upper-anterior and lower-anterior. The four-grade scale to assess each lung field was 0 to 3, where border scores corresponded to the normal lung (“0”) and solid pulmonary consolidations (“3”). The sum of all lung field scores was the overall LUS score. This score demonstrated predictive value with regard to the need for endotracheal intubation before 72 h of life (AUC = 0.804; 95% CI: 0.673–0.935; *p* = 0.001), and the median score for infants requiring intubation was 9 (IQR 8–12) [8].

Lung scans in neonatal RDS exhibit a largely homogenous picture, especially during the first h of life, as demonstrated by Reimondi et al. [9] and in animal immature lung models [10]. Clinical observations, however, show that in the subsequent days of life, pulmonary pathologies in premature neonates often tend to locate in the posterior lung fields [11–13]. This is consistent with the gravitational effect, which affects the lowest parts of the lungs in the supine position. In nonhomogeneous lung disorders, the involvement of posterior lung fields seems even more significant. Examples include meconium aspiration syndrome [14] or neonatal ARDS [15]. Posterior scans, therefore, provide valuable information that should not be overlooked.

Thus, we propose an alternative approach to lung ultrasound assessment that relies, in short, on (1) scanning posterior rather than lateral fields, (2) evaluating anterior fields as a whole, without division into upper and lower parts and (3) introducing an additional grade to the current scoring scheme.

The aim of this study was to confirm the validity of the proposed LUS score in terms of its reproducibility and correlation with blood oxygenation and to assess the ability of early postnatal scans to predict the need for respiratory support on the third day of life (DOL 3). As a secondary analysis, we assessed the weight of posterior vs. anterior field scores in the overall LUS score based on serial scans performed over a period of one month.

## Methods

### Patients and study design

This was a monocentric, prospective cohort study. Serial lung ultrasound scans were performed in 70 premature newborns admitted to the neonatal intensive care unit (NICU) of the Children’s University Hospital in Cracow between January 2013 and March 2015. The study center is an academic, tertiary-referral 30-bed NICU, with approximately 450 admissions per year. The hospital does not have a maternity ward, and all study patients were outborn. Infants with RDS were considered for the study if they were ≤32 weeks of gestation, had a birth weight ≤1500 g and were admitted within the first 24 h of life. RDS was diagnosed based on criteria described in the 2013 European Consensus Guidelines on RDS Management, which included PaO_2_ < 50 mm Hg (< 6.6 kPa) in room air, central cyanosis in room air or the need for supplemental oxygen to maintain PaO_2_ > 50 mm Hg (> 6.6 kPa) [16]. Complementary imaging examinations included either chest X-rays presenting typical lung appearance or lung scans performed by experienced neonatal sonographers. The ultrasound criteria for RDS included (1) bilateral signs of abnormalities of the pleural line (thickened and irregular pleural line), (2) white lung images, and (3) absence of spared areas in all lung fields [17].

The occurrence of major malformations in the neonate was a criterion excluding enrollment in the study. These involved hemodynamically significant heart disease and other organ abnormalities that may adversely impair respiratory function. All parents or legal guardians provided written informed consent, and the study protocol was approved by the Bioethical Committee of Jagiellonian University, Cracow.

### Ultrasound examinations

Newborns meeting the enrollment criteria were subjected to an initial lung ultrasound scan within the first 24 h of life (LUS_0_) and subsequent scans on days 2, 3, 5, 7, 10, 14, 21 and 28. Examinations were performed by two expert-level neonatal sonographers using a Phillips HD 11 scanner with a linear probe of 12 − 5 MHz. Four lung areas were assessed: anterior (left), anterior (right), posterior (left) and posterior (right), using transversal and longitudinal positions of the probe. At least one hour before lung examination, the infant’s position was not changed. If the infant was supine (86% of all scans), LUS was begun with the assessment of the anterior lung fields. Subsequently, posterior fields were assessed after the infant was rotated sideways. If the patient was in the prone position (11% of all scans), the LUS began with scans of posterior fields. The predominance of the supine position resulted from the presence of umbilical catheters especially in the first days of life. In 4% of all scans, the infant’s position was not recorded.

**Fig. 1:**
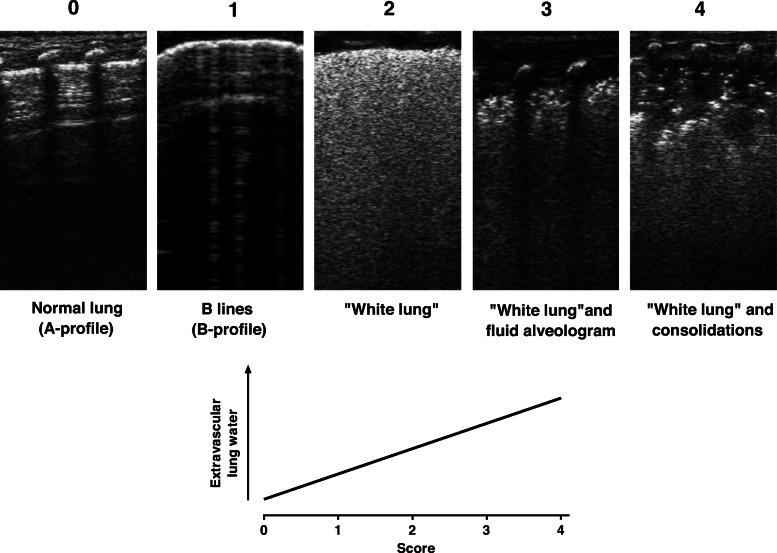
The grading system for the evaluation of pulmonary areas, based on the pleural ultrasound artifacts

Each lung field was graded according to the five-grade scale shown in Fig. 1, where “0” corresponded to normal lung and “4” indicated the presence of pulmonary consolidations. The sum of all four area scores was the total LUS score, which could therefore range from 0 to 16. Bearing in mind that the scale must take into account not only pulmonary changes arising within the first 24 h of birth but also within 28 days, we added an additional grade of “white lung with fluid alveologram” (score “3”). This sonographic pattern has previously been identified and described as a homogenous, highly hypoechoic band found in the subpleural location [18, 19]. The purpose of the introduction of this new category to the LUS score was to achieve a more precise, continuous assessment of the severity of lung lesions. The fluid alveologram represents the transition from white lung to solid consolidation and is readily distinguishable.

All scans were recorded and stored as uncompressed video files on an external storage device for subsequent evaluation of interrater agreement. Each investigator was blinded to the evaluations given by the other rater.

### Study measures

Our primary outcome measures for the validity of the modified LUS score were the correlation coefficient determining the relationship between the LUS score and infant oxygenation status and the estimates of interrater agreement. The ratio of SpO_2_/FiO_2_ (S/F) served as an oxygenation measure against which correlation with the LUS score was assessed.

To evaluate the relationship between the LUS score and ventilation modes, the scores from all scans were plotted vs. concurrent modes of respiratory support. The modes considered included the following interventions (in escalating order): oxygen delivery via nasal cannula (low flow), continuous positive airway pressure (CPAP) and invasive ventilation. The latter was defined as ventilation requiring endotracheal intubation and encompassed standard intermittent positive pressure ventilation (IPPV) and high frequency oscillatory ventilation (HFOV). The criteria for invasive ventilation included recurrent prolonged apneic episodes associated with bradycardia that did not respond to stimulus and/or PCO_2_ > 8 kPa and/or FiO_2_ > 0.6. In our center, HFOV was used as rescue therapy in cases where no stabilization of respiratory function could be achieved with IPPV.

To test for the prognostic capability of the LUS score with regard to the need for subsequent respiratory support, probabilities for various respiratory support modes applied on day of life 3 (DOL 3) were calculated using scans performed in the first 24 h of life (LUS_0_).

The weight of the posterior lung fields in the total LUS score was determined by calculating the least square (ls) means of the scores of the posterior and anterior fields based on all scans performed within the 28-day period.

### Statistics

The association of the LUS score with S/F was evaluated using the correlation coefficient of Spearman ranks. To check for interrater agreement, the intraclass correlation coefficient and Cronbach’s alpha were calculated. Probabilities for the need for particular modes of respiratory support on DOL 3 were assessed with stepwise multivariate ordinal logistic regression. Here, mode of respiratory support was the dependent variable, whereas explanatory variables were those that tested significant in the univariate model. The backward elimination approach was used to simplify the multivariate model by eliminating the variable/-s for which their loss provides the most insignificant deterioration of the model fit. The power of prediction of the LUS_0_ in regard to various ventilation modes on DOL 3 was determined with a multiclass area under the curve as described by Hand and Till (2001) [20]. The ability of LUS_0_ to predict invasive ventilation on DOL 3 was assessed with logistic regression. Quality of prediction was determined with the area under the ROC curve.

To assess the weight of the posterior area scores in the total LUS score, we used linear mixed effects regression. In this analysis, patients were set as a random factor, while the anteroposterior gradient of the LUS and the postnatal day of examination were fixed effects. The obtained least square (ls) means of the posterior and anterior scores were subsequently compared. For all analyses, *p* values less than 0.05 were regarded as significant.

## Results

The study cohort consisted of 70 premature newborns with a median gestational age of 28 weeks (IQR 26–29) and a mean birth weight of 1037 ± 270 g. Baseline characteristics are presented in Table 1. Of the 70 babies, 8 (11.4%) died before discharge, and 6 (8.5%) were transferred to another hospital and were lost to follow-up.

**Table 1:** Basic patients’ characteristics

Variable	
Gestational age (weeks), mean ±SD	27.3 ±2.4
Gestational age categories	
22 – 25 weeks	14 (20%)
26 – 28 weeks	34 (49%)
29 – 32 weeks	22 (31%)
Birth weight (g), mean ±SD	1037 ±270
Male	39 (56%)
Cesarean section	47 (67%)
Prenatal steroids	42 (60%)
Time from birth to first scan (hours)	
Median, IQR	10.7 (7.5 – 15.5)
Range	2.8 – 22.4
Respiratory support at first scan	
None	6 (9%)
Oxygen nasal cannula (low flow)	1 (1%)
nCPAP	29 (41%)
IPPV	34 (49%)
Oxygenation status at first scan	
PaO_2_ (kPa)	7.6 (6.2 – 9.5)
FiO_2_	0.26 (0.21 – 0.40)
SpO_2_ (%)	95 (94 – 98)
Treatment with surfactant	51 (73%)
Comorbidity	
Persistent ductus arteriosus	35 (50%)
Atrial septum defect	8 (11%)
Pneumonia	18 (26%)
Oxygen dependency at 36 weeks PMA	38 (54%)

Unless otherwise stated data are n (%) or median (IQR)

*nCPAP* nasal continuous positive airway pressure, *IPPV* intermittent positive pressure ventilation, *PaO*_*2*_ arterial partial pressure of oxygen, *FiO*_*2*_ fraction of inspired oxygen, *SpO*_*2*_ blood oxygen saturation, *PMA* postmenstrual age

A total of 647 lung sonograms were performed, including 625 (97%) *per protocol* scans and 22 (3%) complementary scans made for clinical indications beyond the day of life 28. In 6 of 647 (0.9%) examinations, LUS was not calculated because scans revealed pneumothorax (*n* = 5) or hydrothorax (*n* = 1). These 6 scans were excluded from the analysis together with the 22 complementary scans. Therefore, analyses were performed based on 619 scans. Baseline sonograms were performed on average 10.7 (7.5–15.5) h after birth (median, IQR).

**Fig. 2:**
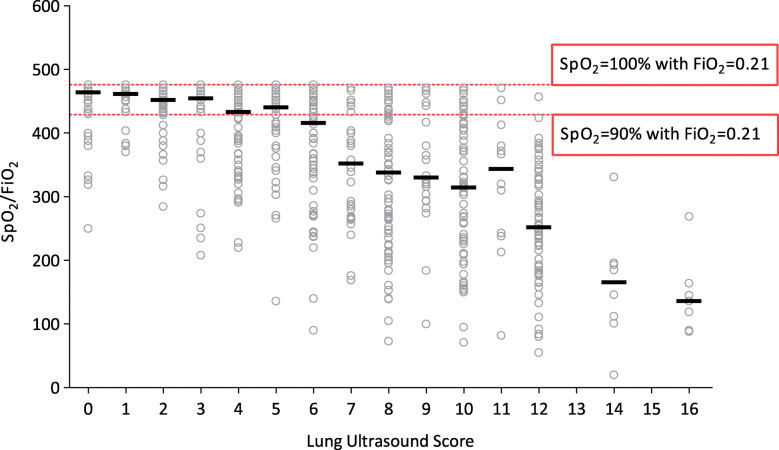
Distribution of LUS and the corresponding SpO_2_/FiO_2_. Horizontal lines depict median SpO_2_/FiO_2_ values

LUS scores correlated significantly with S/F (Spearman rho = −0.635; *p* < 0.001). The distribution of the LUS scores and the corresponding S/F is presented in Fig. 2.

A very high consistency was found between the LUS ratings of both evaluators. An intraclass correlation coefficient equal to 0.94 (95% CI 0.93–0.95) and Cronbach’s alpha equal to 0.99 showed excellent interrater agreement.

**Fig. 3:**
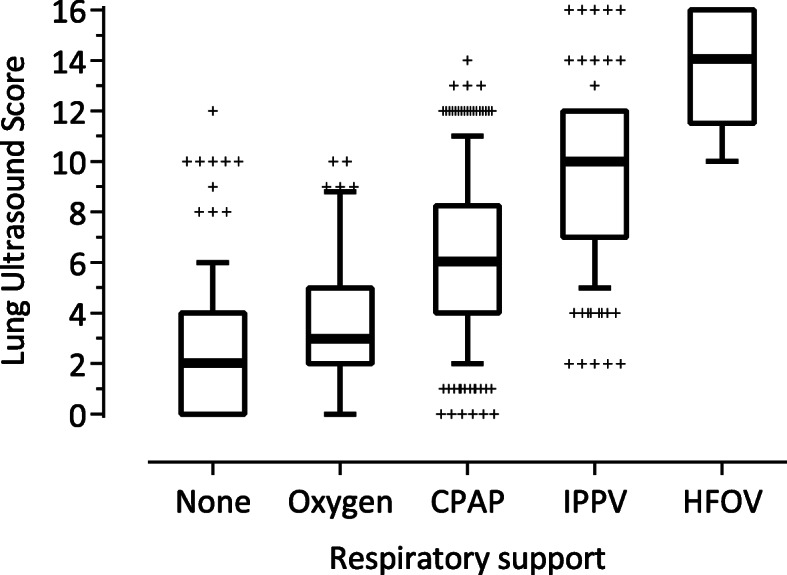
Lung ultrasound score vs. mode of respiratory support. Horizontal lines are medians, boxes denote interquartile range and whiskers are 10th and 90th percentile. Outlying scores are marked with crosses

LUS presented linear growth depending on the requirement for respiratory support. Infants who breathed spontaneously and did not need oxygen supplementation had a median (IQR) LUS of 2 (0–4). Escalation of therapy from oxygen supplementation to CPAP and further to invasive ventilation (IPPV and HFOV as the ultimate option) involved a linear increase in LUS; the respective medians (IQR) were 3 (2-4.5) for O_2_ cannula, 6 (4–8) for CPAP, 10 (7–12) for IPPV and 14 (12.5–15.5) for HFOV (Fig. 3).

In the univariate ordinal logistic analysis of the variables assessed for correlation with the need for invasive ventilation (LUS_0_, birth weight, gestational age, maternal steroids, cesarean section, surfactant therapy), the following variables were significant: LUS_0_ (OR 1.41, 95% CI 1.31–1.51), birth weight (OR 0.43, 95% CI 0.36–0.51), gestational age (OR 0.56, 95% CI 1.31–1.51) and surfactant (OR 6.23, 95% CI 2.48–5.63). In the final stepwise multivariate regression model, the need for invasive ventilation on DOL 3 was predicted only by LUS_0_ (p = 0.016) and birth weight (*p* < 0.0001). LUS_0_ exhibited high reliability in predicting the need for invasive ventilation on DOL 3, as reflected by logistic regression model for which the area under the ROC curve was equal to 0.845 (95% DeLong CI: 0.738–0.951; *p* < 0.001).

**Fig. 4:**
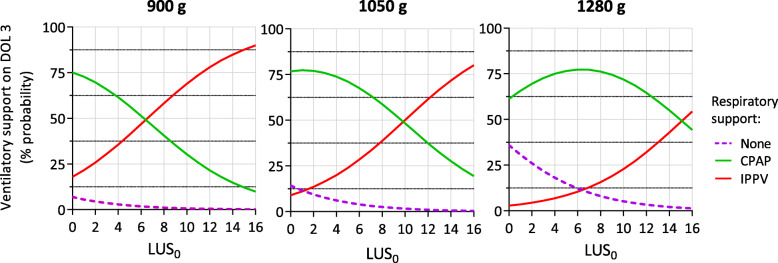
Probability of the need for respiratory support on the third day of life, depending on the LUS performed within 24 h from birth (LUS_0_). Probabilities calculated for the median birth weight (middle graph), 25th percentile (left graph) and 75th percentile (right graph). No infants in the study cohort were using O_2_ cannula or HFOV on DOL 3

The probabilities of various ventilation modes on DOL 3 are presented in Fig. 4. Depending on the birth weight (BW), invasive ventilation was the most likely option of respiratory support on DOL 3 if the following thresholds of LUS_0_ were reached: ≥7 (for BW 900 g), ≥ 10 (for BW 1050 g) and ≥ 15 (for BW 1280 g). The above birth weights are 25th percentile, median and 75th percentile of the cohort studied.

A multiclass area under the curve equal to 0.865 indicated high power of prediction of various respiratory support modes with LUS_0_.

The posterior pulmonary field scores were significantly higher compared with the anterior field scores. Based on all scans performed, the posterior fields had an average score (ls mean, confidence level) of 4.0 (3.8–4.1), whereas anterior pulmonary fields had an average score of 2.2 (2.0–2.4). The difference was statistically significant (1.8; *p* < 0.001).

## Discussion

To date, several studies have been published assessing the clinical usefulness of lung ultrasound in newborns with respiratory disorders [1–7, 21–27]. These works focused mainly on preterm infants with RDS and transient tachypnea of the newborn; in both cases, LUS was performed primarily on the first day of life. [1–7, 21–24, 26, 27].

Our research provides an additional body of evidence, as it was based on post-birth lung sonograms complemented by sequential scans performed over a period of 28 days. As a result, the correlation of the LUS score with the severity of respiratory failure as expressed by S/F and the need for respiratory support was demonstrated beyond the first day of life. It must be noted, however, that the S/F, which we used as an oxygenation measure, is an imperfect parameter that can be affected by other factors, such as HbF, peripheral perfusion, temperature, or persistent ductus arteriosus. Arterial oxygen tension would be a more reliable parameter. Unfortunately, PaO_2_ measurements were only obtained in the first days of life when the umbilical catheters were in use and were not available for later scans.

Our study covered a diverse spectrum of patients, both before and after administration of surfactant, infants with significant posttreatment improvement and those who deteriorated in the follow-up examinations, patients with developing pneumonia and those with bronchopulmonary dysplasia. Sonograms were obtained from patients who represented all sorts of respiratory statuses, ranging from spontaneously breathing ambient air through nasal CPAP to invasive ventilation. Thus, a close correlation between the LUS and S/F, which is a marker of respiratory failure, was demonstrated in much more diverse clinical conditions. This allows the LUS to be treated as a more universal measure of pulmonary pathology, not limited to the typical changes observed in neonates with RDS on the first day of life. On the other hand, such heterogeneity of the clinical status under which the scans were performed can also be considered a limitation, which hinders the standardization of the results.

In studies where lung ultrasound images were quantified, four-grade scales were mainly used (a score of 0-1-2-3) [1, 3, 5, 6, 21–24]. In our work, we proposed a five-grade scale (a score of 0-1-2-3-4), defining an additional category of “white lung with fluid alveologram”. This sonographic pattern is sometimes referred to in the literature as *superficial consolidations* and should be distinguished from true consolidations [18, 19]. True consolidations are hypoechogenic triangular-shaped changes with visible air bronchogram, while fluid alveologram is a subpleural accumulation of exudative fluid, which mixed with air, fulfills the alveoli. A fluid alveologram can be observed in severe RDS [23] and, as a rule, is not seen in the first day of life but rather in the following days. Since the study covered the first 28 days of life of premature babies, it seemed appropriate to classify this type of change separately.

We abandoned the division of pulmonary fields into upper and lower sections. Previously, Pang et al. [23] expanded the assessment scales by including posterior pulmonary fields, dividing each of the studied lung areas into upper and lower parts while maintaining a four-grade scale. This type of division used also by Raimondi et al. [24] is adopted from adult studies, where due to the chest size, it seems expedient.

In almost all available works, ultrasound examination was limited to the anterior and lateral parts of the lungs. The “anterior-lateral” approach does not take full advantage of lung ultrasound over standard anteroposterior X-ray, namely, the possibility of distinguishing between changes in the anterior and posterior parts of the lungs. Very few studies have reported assessments of posterior pulmonary fields [4, 23, 24]. In our modification of the lung ultrasound examination, we propose assessment of posterior and not lateral pulmonary fields. In supine infants, examination of the posterior fields was performed after the patient’s rotation to the side. As the examination was short at approximately 30 s, it did not cause problems or destabilize the patient’s condition.

It is known from daily clinical practice that many pulmonary pathologies tend to have gravitational positioning. Our study confirmed this observation, as a review of serial lung scans performed over a period of 28 days demonstrated that the scores of the posterior pulmonary fields were significantly higher than the anterior field scores (ls means 4.0 vs. 2.2; *p* < 0.001). We used ls means to compare posterior vs. anterior scores given that, contrary to regular (arithmetic) means, ls-means are based on a linear mixed-effect model and thus adjust for confounding effects, which were repeated examinations in each patient in this case.

In previous reports, the LUS score was found to predict various clinical outcomes in neonates with RDS, including the need for surfactant treatment [5, 8, 28], admission to the neonatal intensive care unit [29] or histological lung injury [30].

In our work, we demonstrated the significant predictive ability of the postnatal (performed within 24 h from birth) LUS score to predict the need for mechanical ventilation on DOL 3. The need for invasive ventilation on DOL 3 is a widely recognized endpoint in neonatal RDS and is strongly correlated with subsequent adverse outcomes [31–33]. Depending on the birth weight, specific LUS cut-off levels indicate infants in whom invasive ventilation is the most likely mode of respiratory support on DOL 3. Thus, our findings may have significant practical implications. Modified LUS allows for early identification of the most vulnerable infants and may lead to earlier decisions, e.g., administration of exogenous surfactant or more aggressive respiratory support.

The analysis also provides an important observation regarding the association of oxygen requirements and the severity of lung changes. As shown in Fig. 2, the increasing severity of pulmonary changes is not accompanied by a parallel increase in oxygen demand until the LUS score reaches 5–6 points, which corresponds to approximately 1/3 of the LUS maximum possible score. Simply put, this means that only when approximately one-third of the lung parenchyma is involved in the pathological process does an increased demand for oxygen appear. This observation is not surprising from a pathophysiological point of view, as for most organs (kidney, liver), the pathological process must cover a certain area of ​​the organ to cause clinical symptoms [34].

However, this fact sheds new light on the applied criteria for surfactant treatment. According to the current therapeutic guidelines, the decision of surfactant administration is based on the required level of inspired oxygen [35]. With the current approach, if there is no increased oxygen requirement, surfactant is not applied. In light of our findings, this means that treatment is not administered until the severity of lung changes as measured by LUS sore reach at least 1/3 of its maximum possible level. The current treatment paradigm requires verification in properly designed clinical trials where surfactant would be LUS guided. Papers describing the use of the LUS score as a criterion for the administration of surfactant have already emerged, presenting clinical benefits of this novel approach, e.g., an increased number of ventilator-free days [21] or reduced oxygen exposure [36], while maintaining unchanged pharmaceutical expenditures [37]. It is essential that in future studies, the primary outcome is not CPAP failure, as it is at present, but the development of bronchopulmonary dysplasia.

In our study, LUS assessments had a very high interrater agreement, as reflected by the intraclass correlation coefficient and Cronbach’s alpha. Although all scans were assessed by two neonatologists with expert-level skills, lung ultrasound is known to be a diagnostic procedure that is easy to master and has a steep learning curve. Daily observations outside the scope of this study show that lung ultrasound examination can be easily learned during two-day courses and 1–2 months of supervised practice. It is therefore likely that the reproducibility of the LUS assessments carried out by ultrasound experts would be replicated by less experienced sonographers.

The current situation of using different scales for assessing the severity of changes in lung ultrasound in newborns is summarized in a meta-analysis published by Razak et al. [38]. In their conclusions, the authors emphasize the close correlation of ultrasound assessment to clinical parameters, which proves the usefulness of this tool.

## Conclusions

In summary, we proposed a modified LUS, which is characterized by a high correlation with oxygenation parameters and respiratory support modes and a very high consistency of assessments between performers. We also demonstrated the importance of the posterior fields in ultrasound assessment of the lungs. The study showed that LUS is a tool that can be used for the early postnatal identification of infants at risk of invasive ventilation in the subsequent days of life. Finally, the modified LUS is suitable not only in newborns during the first days of life and remaining on noninvasive respiratory support but also in mechanically ventilated infants with various respiratory disorders during the first month of life.

## Data Availability

The datasets analyzed during the current study are available from the corresponding author on reasonable request.

## Abbreviations

LUS: Lung ultrasound
RDS: Respiratory distress syndrome
DOL: Day of life
S/F: Oxygen saturation over fraction of inspired oxygen
ICC: Intraclass correlation coefficient
CI: Confidence interval
ROC: Receiver operating characteristic
AUC: Area under curve
ARDS: Acute respiratory distress syndrome
NICU: Neonatal intensive care unit
CPAP: Continuous positive airway pressure
IPPV: Intermittent positive pressure ventilation
HFOV: High frequency oscillatory ventilation
FiO_2_: Fraction of inspired oxygen
PMA: Postmenstrual age
SpO_2_: Blood oxygen saturation
HbF: Fetal hemoglobin
OR: Odds ratio
BW: Birth weight
